# Improving clinical competency using simulation technology

**DOI:** 10.1097/01.NURSE.0000668448.43535.4f

**Published:** 2020-07

**Authors:** LLYNNE C. KIERNAN, DARLENE M. OLSEN

**Affiliations:** Norwich University in Northfield, Vt.

TODAY’S HIGHLY demanding healthcare environment requires effective critical thinking and clinical skills for optimal patient outcomes in complex care situations. To bridge the gap between nursing students and newly graduated nurses, the nursing faculty must foster clinical skills acquisition to avoid medical errors. Using simulation technology allows nursing students to apply knowledge and skills to transfer theory into clinical practice.^1^ This article discusses a study to assess the perceived competency of junior and senior nursing students in clinical behaviors and skills.

## Background

Newly graduated nurses enter the clinical setting with little experience and many expectations placed upon them. Proficiency in basic clinical skills, critical thinking, and healthcare decision-making have been identified as gaps that need improvement among these nurses.^2^ The greatest areas for improvement among recent nursing graduates include safe medication administration; failure to rescue; patient falls; risk management; and multitasking, prioritizing, and delegating responsibilities.^2^ Although these nurses may enter clinical settings with extensive theoretical knowledge, procedural competence is critical for a successful transition into practice.^3^

Medical errors represent the third leading cause of death in hospitals, and school of nursing (SON) teaching faculties must foster clinical competence to reduce errors and promote positive patient outcomes.^4^ In response to the changing needs of patients and the complexities of nursing practice, a transformation in nursing education is necessary.^5^

Educators can use simulation technology to observe and document nursing students’ competence in clinical skill acquisition. *Deliberate practice* is defined as activities to improve performance.^6^ It includes feedback on students’ actual performance compared with their desired performance, providing opportunity for repetition until they achieve their educational goals.^6^ This feedback facilitates the development of sound clinical practice habits.

To date, no best-practice model for simulation training is available and debriefing is often disregarded in research.^1^ In the study described below, the authors used the clinical competency questionnaire (CCQ) to assess improvements in clinical competence among junior and senior nursing students before and after simulation training and clinical practicum experience.^7^

## Methods

The study utilized a pre- and posttest design and received institutional review board (IRB) approval from the authors’ university. Participants completed an IRB-approved consent form before the data collection process. Participation was voluntary, and the participants could discontinue the study at any time. The purpose and procedures were explained to the participating nursing students, and any questions were addressed. The authors collected demographic information on age, gender, and ethnicity, and all data were paired and de-identified to maintain confidentiality.

Including all class levels, 145 students were enrolled in the baccalaureate nursing program at a private university accredited by the New England Association of Schools and Colleges and the Commission on Collegiate Nursing Education. Only junior- and senior-year students were invited to participate. There were 27 first-semester juniors and 35 first-semester seniors; all 62 participated in basic and advanced skills training in the simulation and clinical skills labs with the use of digital tablets.

The average age of participants in their junior year was 23.9. There were 23 females and 4 males, and 21 participants identified as White. The average age of participants in their senior year was 26 years, with 28 females and 7 males. Thirty-one participants identified as White. All participants were English-speaking.

During the 15-week semester, junior-year nursing students were enrolled in the Medical-Surgical Nursing I clinical practicum and placed in various rural hospital settings and one rehabilitation facility. Seniors were enrolled in the Medical-Surgical Nursing II clinical practicum and placed in one of two university hospitals for the semester.

The CCQ measured the students’ self-assessments of professional nursing behaviors and perceived clinical competencies.^3,7^ This 47-item questionnaire was developed and psychometrically analyzed by Liou and Cheng and demonstrates good reliability and validity.^7^ Items 1 through 16 focused on professional nursing behaviors (see CCQ items 1–16), while items 17 through 47 focused on skills competencies (see CCQ items 17–47).

Cronbach’s alpha reports the internal consistency reliability of a multicomponent scale to determine consistency in measuring the same attribute. It can range from 0 to 1, and values closer to 1.00 demonstrate stronger evidence of reliability. Values of 0.70 or higher are considered acceptable.^8^ In total, the CCQ had a Cronbach’s alpha of 0.98 and a mean item-total correlation of 0.70 (range: 0.50 to 0.81). The professional nursing behaviors category had a Cronbach’s alpha of 0.95 and a mean item-total of 0.73; the skills competency category had a Cronbach’s alpha of 0.97 and a mean item-total of 0.72.^7^

## Procedure and simulations

At the start of the study, nursing students completed the 47-item CCQ to self-assess their clinical abilities. Each item was scored on a Likert scale of 1 to 5, with 1 representing complete unfamiliarity and 5 representing theoretical understanding and competency in practice without supervision. The faculty at the authors’ SON developed a consensus of basic and advanced skills and determined that the items on the CCQ fit the context for this study. They utilized educational sessions, demonstrated clinical skills, and observed and documented student demonstrations of clinical skills. Video recordings using digital tablets were utilized to observe performance for student self-evaluations and specific, comprehensive faculty debriefing.^9^

Professional nursing behaviors included adhering to health and safety precautions, maintaining patient confidentiality, and applying critical thinking; skills competencies included medication administration, sterile procedures, nasogastric tube placement, and chest tube care. Nursing students were required to demonstrate clinical competency, and the simulation lab was opened for practice and supervised by the faculty and simulation coordinator. The simulation scenarios and debriefing evaluations were integrated into the clinical curriculum for the Medical-Surgical I and II courses during the semester.

Task trainers, as well as manikin-based simulation and high-fidelity simulations, were used to develop clinical skills. Task trainers represent part of the human body, such as an arm or abdomen, and use mechanical or electronic interfaces to teach and give feedback on procedural skills training. Manikin-based simulations use manikins to represent patients, including heart and lung sounds, palpable pulses, and voice interaction using computer software. High-fidelity evidence-based simulation scenarios, which provide highly realistic and interactive learning experiences, were developed specifically for the junior and senior nursing students to incorporate the skills tested by the CCQ.^10^

Simulation scenarios for the junior nursing students included caring for a patient with hypoxemia, a patient with anaphylaxis, and a postoperative cholecystectomy patient with diabetes. Scenarios for senior nursing students included caring for patients with autonomic dysreflexia, acute transfusion reactions, and ST-elevation myocardial infarctions. Each group had four to five participants taking on different roles to care for the simulated patient, with clinical roles changing in each scenario.^11^

The simulations lasted 35 minutes, followed by 40 minutes of debriefing. Video debriefings were recorded to validate psychomotor skill performance and provided opportunities to fine-tune deliberate practice.^12^ Repetitive practice augmented with innovative technology and faculty feedback was embedded into the high-fidelity simulator, allowing students to acquire, enhance, and reflect on their skills competencies.^10,13^ The CCQ was administered again after participants had undergone simulations.

## Data analysis and results

Pre- and posttest results were recorded, and data were analyzed using open source statistical software.^14^ A Wilcoxon signed rank test is a statistical hypothesis test for paired data generated on a Likert scale.^15,16^ It was used to identify the difference between pre- and posttest rankings and determine improvements in self-assessed clinical competencies after the simulation intervention. Each CCQ question was analyzed individually by class year using a significance level of 5%. The students’ clinical practicum site had no significant effect on results.

Scores related to professional nursing behaviors demonstrated a statistically significant improvement (*P* = less than .05) for most items in both the junior and senior groups (see CCQ summary statistics). Neither group demonstrated a significant improvement in professional attire, as the item ranked high in both the pre- and posttest scores. Both groups saw a large improvement in taking precautions to minimize risks to patients.

The largest improvement for juniors addressed critical thinking and accepting constructive criticism. The seniors demonstrated largest improvement in understanding the legal and ethical rights of patients. They also improved in self-assessments of communication skills, but this was not to a statistically significant degree. For the junior participants, the combined rankings of professional competencies improved from the pretest (mean [M] = 3.62; standard deviation [SD] = 0.87) to the posttest (M = 4.32; SD = 0.71). On the other hand, the senior participants demonstrated similar improvements from the pretest (M = 4.18; SD = 0.71) to the posttest (M = 4.61; SD = 0.59).

Senior scores related to nursing skills competencies also demonstrated statistically significant improvements in each CCQ item (*P* = less than .05). They had the largest improvements in I.V. medication administration and tracheotomy and chest tube care. Their combined competence rankings improved from the pretest (M = 3.6; SD = 0.95) to the posttest (M = 4.2; SD = 0.89).

Among junior-year nursing students, skills competencies showed slightly less improvement between the pre- (M = 2.99; SD = 1.02) and posttest (M = 3.41; SD = 1.07). Their competence improved in general, particularly inpatient assessment and care plans, but they did not show significant improvement in 8 of the 47 items on the CCQ. This was expected in seven of the eight items, as these were newly introduced skills for these students, but urinary catheter insertion and care also demonstrated a downward trend in competence. The students had been instructed in this skill in their sophomore year and it had been reviewed in the simulation scenarios, but competence still decreased between the pre- (M = 3.26; SD = 0.66) and posttest (M = 3.15; SD = 0.86). (See Item 42: Urinary catheter insertion and care.)

## Discussion

This study demonstrated that standard instruction and interventions are not fostering competence in urinary catheter insertion and care among nursing students, despite being a procedural skill required for graduation.^3^ Catheterization of the urinary tract is a precipitating cause of hospital-acquired catheter-associated urinary tract infections (CAUTIs), resulting in more than 13,000 deaths and an estimated cost of more than $340 million annually.^17,18^ Newly graduated nurses who are not yet proficient in urinary catheter insertion and care may cause iatrogenic CAUTIs secondary to poor technique. In addition to negative patient outcomes, hospital reimbursement for the subsequent care of these patients may be decreased or denied.

## Summary

Quantitative assessment was used in nursing students’ evaluations of their own competence before and after simulation training. The data showed areas of significant improvement, areas of continued competence, and areas of declined self-reported competence. This suggests the need for increased assistance and supervision from nursing faculty.

Deliberate practice and video debriefing were effective modalities in simulation technology for nursing skills acquisition and self-assessment. Nursing education develops knowledgeable nurses who are capable of providing safe, highly competent, and skilled patient care. Skills in which students may lack competence, such as urinary catheter management, may improve with additional instruction.

However, the study also had several limitations. The data were collected from a convenience sample of junior and senior baccalaureate nursing students in a traditional nursing program from one university. As such, the results may not be generalized to other nursing programs, such as RN-to-BSN programs or accelerated BSN programs. Additionally, the CCQ is a self-assessment tool designed to measure only the perceived competence of professional nursing behaviors and skills rather than practical competence, so it was more subjective.^3^

## Figures and Tables

**Figure:**
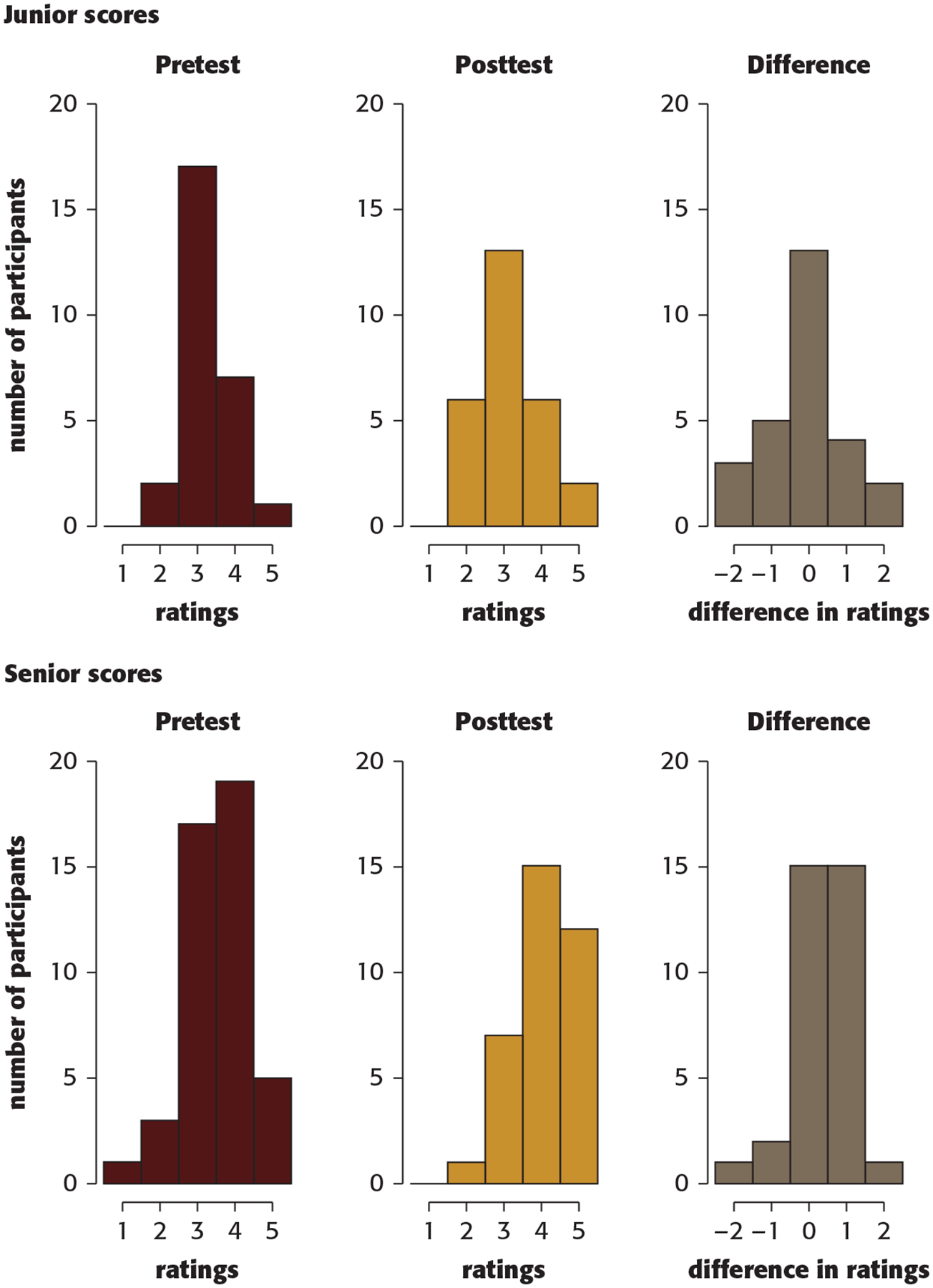
Item 42: Urinary catheter insertion and care Twenty-one juniors (77.7%) had either a decrease or no improvement in self-assessment ratings of urinary catheter insertion and care. A Wilcoxon signed-rank test (W) indicated no significant improvement in the juniors’ scores, despite previous education (W = 44, *P* = .721). In contrast, the seniors demonstrated a significant upward trend in self-assessment ratings for this task (W = 173.5, *P* = .003).

**Table:** CCQ items 1–16^7^ The CCQ measured the students’ self-assessments of professional nursing behaviors and perceived clinical competencies. At the start of the study, nursing students completed the 47-item CCQ to self-assess their clinical abilities. The entire CCQ was administered again after participants had undergone simulations. Below are the first 16 items, which are related to professional nursing behaviors.

Professional nursing behaviors		1	2	3	4	5
**1.**	Following health and safety precautions					
**2.**	Taking appropriate measures to prevent or minimize risk of injury to self					
**3.**	Taking appropriate measures to prevent or minimize risk of injury to patients					
**4.**	Preventing patients from problem occurrence					
**5.**	Adhering to the regulation of patients’ and families’ confidentiality					
**6.**	Demonstrating cultural competence					
**7.**	Adhering to ethical and legal standards of practice					
**8.**	Maintaining appropriate appearance, attire, and conduct					
**9.**	Understanding patient rights					
**10.**	Recognizing and maximizing opportunity for learning					
**11.**	Applying appropriate measures and resources to solve problems					
**12.**	Applying or accepting constructive criticism					
**13.**	Applying critical thinking to patient care					
**14.**	Communicating verbally with precise and appropriate terminology in a timely manner with patients and families					
**15.**	Communicating verbally with precise and appropriate terminology in a timely manner with healthcare professionals					
**16.**	Understanding and supporting group goals					

**Key:** 1. Do not have a clue 2. Know in theory, but not confident at all in practice 3. Know in theory, can perform some parts in practice independently, need supervision to be readily available 4. Know in theory, competent in practice, need contactable sources of supervision 5. Know in theory, competent in practice without supervision.

Adapted with permission from: Liou SR, Cheng CY. Developing and validating the clinical competence questionnaire: a self-assessment instrument for upcoming baccalaureate nursing graduates. *J Nurs Educ Pract*. 2014;4(2):56–66.

**Table:** CCQ items 17–47^7^ Below are items 17 through 47 of the CCQ, which are related to skill competencies.

Skill competencies		1	2	3	4	5
**17.**	Performing and documenting patient health assessment					
**18.**	Answering questions for patients or families					
**19.**	Educating patients or families with disease-related care knowledge					
**20.**	Charting and documentation					
**21.**	Developing care plan for patients					
**22.**	Performing shift report using situation, background, assessment, and recommendation communication					
**23.**	Performing hygiene and daily care routines					
**24.**	Providing rest and comfort measures					
**25.**	Assessing nutrition and fluid balance					
**26.**	Assessing elimination					
**27.**	Assisting activities and mobility, and changing position					
**28.**	Providing emotional and psychosocial support					
**29.**	Changing I.V. fluid bottle or bag					
**30.**	Administering secondary I.V. antibiotic					
**31.**	Administering I.M. and Z-track medications					
**32.**	Performing subcutaneous injection					
**33.**	Administering oral medications					
**34.**	Performing sterile technique					
**35.**	Performing postural drainage and percussion, and providing oxygen therapy					
**36.**	Performing nasogastric tube feeding and care					
**37.**	Performing wound dressing care					
**38.**	Performing venipuncture					
**39.**	Starting I.V. injections (initiating peripheral venous access)					
**40.**	Administering I.V. medications (or into I.V. bags)					
**41.**	Administering blood transfusion					
**42.**	Performing urinary catheter insertion and care					
**43.**	Performing pre- and postoperative care					
**44.**	Performing an enema					
**45.**	Performing upper airway suction					
**46.**	Performing tracheotomy care					
**47.**	Performing chest tube care with underwater seal management					

**Key:** 1. Do not have a clue 2. Know in theory, but not confident at all in practice 3. Know in theory, can perform some parts in practice independently, need supervision to be readily available 4. Know in theory, competent in practice, need contactable sources of supervision 5. Know in theory, competent in practice without supervision.

Adapted with permission from: Liou SR, Cheng CY. Developing and validating the clinical competence questionnaire: a self-assessment instrument for upcoming baccalaureate nursing graduates. *J Nurs Educ Pract*. 2014;4(2):56–66.

**Table:** CCQ summary statistics Below are pre- and posttest statistics by student class level.

	Juniors (*n* = 27)		Seniors (*n* = 35)	
	Pretest	Posttest	Pretest	Posttest
**Professional**	M = 3.62	M = 4.32	M = 4.18	M = 4.61
**nursing behaviors**	SD = 0.87	SD = 0.71	SD = 0.71	SD = 0.59
**Skill competencies**	M = 2.99	M = 3.41	M = 3.62	M = 4.16
	SD = 1.02	SD = 1.07	SD = 0.95	SD = 0.89

Key: M = mean; SD = standard deviation
